# Strategies to Combat Antibiotic Resistance in the Wastewater Treatment Plants

**DOI:** 10.3389/fmicb.2017.02603

**Published:** 2018-01-17

**Authors:** Fateme Barancheshme, Mariya Munir

**Affiliations:** Department of Civil and Environmental Engineering, University of North Carolina at Charlotte, Charlotte, NC, United States

**Keywords:** antibiotic resistant genes, antibiotic resistant bacteria, treatment strategies, wastewater treatment, nanomaterial, coagulation, biochar, disinfection

## Abstract

The main goal of this manuscript is to review different treatment strategies and mechanisms for combating the antibiotic resistant bacteria (ARB) and antibiotic resistant genes (ARGs) in the wastewater environment. The high amount of antibiotics is released into the wastewater that may promote selection of ARB and ARGs which find their way into natural environments. Emerging microbial pathogens and increasing antibiotic resistance among them is a global public health issue. The propagation and spread of ARB and ARGs in the environment may result in an increase of antibiotic resistant microbial pathogens which is a worldwide environmental and public health concern. A proper treatment of wastewater is essential before its discharge into rivers, lake, or sewage system to prevent the spread of ARB and ARGs into the environment. This review discusses various treatment options applied for combating the spread of ARB and ARGs in wastewater treatment plants (WWTPs). It was reported that low-energy anaerobic–aerobic treatment reactors, constructed wetlands, and disinfection processes have shown good removal efficiencies. Nanomaterials and biochar combined with other treatment methods and coagulation process are very recent strategies regarding ARB and ARGs removal and need more investigation and research. Based on current studies a wide-ranging removal efficiency of ARGs can be achieved depending on the type of genes present and treatment processes used, still, there are gaps that need to be further investigated. In order to find solutions to control dissemination of antibiotic resistance in the environment, it is important to (1) study innovative strategies in large scale and over a long time to reach an actual evaluation, (2) develop risk assessment studies to precisely understand occurrence and abundance of ARB/ARGs so that their potential risks to human health can be determined, and (3) consider operating and environmental factors that affect the efficiency of each treatment mechanism.

## Introduction

### Environmental impact

Recently, World Health Organization (WHO) announced that antibiotic resistance is growing, and we are fast running out of treatment options (Lawe-Davies and Bennett, 2017). Antibiotics serve as selective pressure and the development of antibiotic resistant bacteria (ARB) is linked with the type of antibiotic and the bacterial species (Kolár et al., 2001) therefore measuring the concentration of antibiotics in wastewater, the effluent of WWTPs, and natural water is important. The concentrations of various antibiotics in an effluent-receiving river in Beijing China were characterized where samples were collected from the upstream before the WWTP and the downstream after the WWTP. It was observed that the concentration of tetracycline in the downstream of river was equal to the effluent of WWTP and the concentration of total sulfonamides in the effluent was around 2-fold higher than in the receiving river water suggesting that the effluent containing antibiotics are contaminating the natural water bodies (Table 1; Xu et al., 2015). In another study in Spain occurrence of nine antibiotics was measured at different points to assess the effect of hospitals and WWTPs effluents on a river. In this study, antibiotics, namely ofloxacin, azithromycin, trimethoprim, and metronidazole, were detected at high concentrations in downstream river samples (up to 131.0 ng/L for ofloxacin) with no detection in upstream of the WWTP discharge, and ciprofloxacin and sulfamethoxazole showed around ten-fold higher concentrations in downstream rather than in upstream samples (Rodriguez-Mozaz et al., 2015). These studies suggest that discharge of antibiotic through effluent into the natural environment can lead to selective pressure for the occurrence of antibiotic resistance.

**Table 1:** Antibiotics concentrations in the WWTP and receiving river (Xu et al., 2015).

**Site Location**	**Tetracyclines (ng L^−1^)**	**Sulfonamides (ng L^−1^)**	**Quinolones (ng L^−1^)**
WWTP influent	1615.8	2263.0	3664.0
WWTP effluent	195.0	2001.0	3866.0
Upstream	265.2	648.1	728.8
Downstream	345.1	1111.0	2769.0
**Removal efficiency**	**87.9%**	**11.6%**	**Increased**^a^

a *The release of adsorbed Ofloxacin from sludge or suspended particles may contribute to the high level of quinolones at this site*.

The abuse of antibiotics for a long time has resulted in multi-resistant bacteria which carry multiple resistance genes (Icgen and Yilmaz, 2014; Lv et al., 2015; Xu et al., 2017). Multiple drug resistant pathogens are emerging with alarming rate. On September 20, 2017, WHO announced the antibiotic-resistant infections as the greatest risk to health, expected to reach a time in future when people fear common infections and threat their lives from minor surgery. It is reported that drug-resistant tuberculosis kills around 250,000 people each year (WHO, 2017). Antimicrobial resistance claims 25,000 lives in Europe and 23,000 in the US every year (Sachdeva et al., 2017). ARB cause serious disease, for example, methicillin-resistant *Staphylococcus aureus* (MRSA) can cause skin infections (a pimple, impetigo, and scalded skin syndrome), pneumonia, endocarditis, and toxic shock syndrome. Other ARB that can cause life-threatening disease are vancomycin-resistant *Enterococcus*, multi-drug-resistant *Mycobacterium tuberculosis*, and carbapenem-resistant *Enterobacteriaceae gut* bacteria. WHO scientists in July 2017 warned that antibiotic-resistant gonorrhea is growing and gonorrhea can cause very serious complications and sometimes is impossible to treat. Drug-resistant *Salmonella* species are a serious problem for public health worldwide (Su et al., 2004). The emergence of multiple drug resistant pathogenic species are even more problematic. Antibiotic resistance challenge is getting worse while new antibiotics exploration is decreasing, hence it is possible that there would be no defenses against infection in the future (McKinney and Pruden, 2012).

Increasing levels of ARB carrying antibiotic resistance genes (ARGs) in the environment, especially in water and wastewater is a human health issue (Rizzo et al., 2013; Devarajan et al., 2015; Sharma et al., 2016; Li H. et al., 2017). Diverse ARGs have reduced susceptibility of pathogens to different antibiotics like sulfonamide (*sul*), tetracycline (*tet*), fluoroquinolone (*qnr*), macrolide (*erm*), chloramphenicol (*cml, flo*), methicillin (*mec*), and b-lactam (*bla*). High concentrations of ARB and ARGs in industrial, community, clinical, and farming wastewaters are threats to the ecosystems (Devarajan et al., 2015). Surprisingly, based on a study in China, the total ARGs and ARB concentrations in sludge from hospitals were 3 to 4 orders of magnitude higher in residential area samples (Li et al., 2015).

A study on a WWTP using activated sludge and chlorination in their treatment trail showed that the concentration of total tetracycline resistance genes was 6.4 × 10^5^ copies/mL in biosolids and 6.4 × 10^3^ copies/mL in the effluent (Al-Jassim et al., 2015). In another study, at WWTP which applied activated sludge, chlorination and UV irradiation, the concentrations of *tet*(Q) and *tet*(G) in biosolid and effluent was reported to be 2.2 × 10^9^ and 3.4 × 10^4^ copies/mL, respectively. In addition, the copies of resistance genes normalized to the number of bacterial 16S rRNA genes at different sites of a natural river (Cache La Poudre River) ranged from 10^−7^ to 10^−3^ for ARGs [*sul*(1), *sul*(2), *tet*(W), and *tet*(O)](Pei et al., 2006). Therefore, the concentration of ARGs in the effluent of WWTPs is often more than the concentration of ARGs in the natural rivers, and discharge of WWTPs' effluent in the natural rivers lead to dissemination of ARGs in the environment. The study of ARGs occurrence in sediments, lakes, rivers, and soils prove this correlation (Sharma et al., 2016). In another study conducted by Xu et al. (2015) on a river that received the effluent of a WWTP and it was shown that the measured ARGs in the river were identical as found in the WWTP including nine tetracycline resistance genes, four sulfonamide resistance genes, and six quinolone resistance genes (Xu et al., 2015). Additionally, in another study, microbial analysis of water samples collected from 12 stations along Kizilirmak river showed that all the isolates have the multi antibiotic resistant ability. Resistance to aztreonam (63%), pefloxacin (54%), trimethoprim-sulfamethoxazole (54%), gentamicin (50%), oxacillin (46%), penicillin (38%), piperacillin (38%), and ampicillin (38%) were very common (Icgen and Yilmaz, 2014).

On February 2017, WHO published its first ever list of antibiotic-resistant “priority pathogens.” The list includes three classes sorted by the urgency (critical, high and medium priority) with which new antibiotics are needed. Overall, 12 families of bacteria are the greatest threat to human health, as shown in Table 2.

**Table 2:** WHO priority pathogens that need new antibiotics (Lawe-Davies and Bennett, 2017).

**Bacteria**	**Antibiotic resistance**
**PRIORITY 1: CRITICAL**	
*Acinetobacter baumannii*	Carbapenem
*Pseudomonas aeruginosa*	Carbapenem
*Enterobacteriaceae*	Carbapenem, ESBL^a^-producing
**PRIORITY 2: HIGH**	
*Enterococcus faecium*	Vancomycin
*Staphylococcus aureus*	Methicillin, vancomycin-intermediate, and resistant
*Helicobacter pylori*	Clarithromycin
*Campylobacter* spp.	Fluoroquinolone
*Salmonellae*	Fluoroquinolone
*Neisseria gonorrhoeae*	Cephalosporin, fluoroquinolone
**PRIORITY 3: MEDIUM**	
*Streptococcus pneumoniae*	Penicillin-non-susceptible
*Haemophilus influenzae*	Ampicillin
*Shigella* spp.	Fluoroquinolone

a *Extended Spectrum Beta-Lactamases. The ESBL enzyme breaks down and destroys most antibiotics causing them to be inactive, which is why they are not effective against infections caused by these types of bacteria*.

### Sources of ARGs

A low-level antibiotic resistance can occur via natural selection, however, the high level of ARB and ARGs in the environment is due to human activities. ARGs enter the environments from various sources, mainly human and animal sources. WWTPs are among the main anthropogenic sources for occurrence and spread of ARGs while land applications of manure are animal sources (Kemper, 2008; Rizzo et al., 2013). WWTPs receive the discharges from various sources and are hotspots for ARGs that are associated with clinical pathogens (Gao et al., 2012; Pruden et al., 2012; Riquelme et al., 2013; Devarajan et al., 2015; Di Cesare et al., 2016). Many studies demonstrated the presence of ARGs in wastewater that are associated with clinical pathogens (Tseng et al., 2009; Huerta et al., 2013; Rizzo et al., 2013; Devarajan et al., 2015; von Wintersdorff et al., 2016). These ARGs can move along the water cycle by means of wastewater discharge into other aquatic environments (Rizzo et al., 2013). Aquatic ecosystems are ideal sites for occurrence and spread of ARGs since they are constantly polluted by antimicrobial compounds resulting from anthropogenic activities (Rodriguez-Mozaz et al., 2015).

The genetic reactor is the term given to the places where genetic evolution occurs frequently and possibly evolving antibiotic resistance. There are four major places where the genetic evolution occurs frequently and antibiotic resistance evolves; 1. Human and animal microbiota, 2. Hospitals and longstanding care facilities, 3. Wastewater and any form of biological residues and 4. Soil and the surface or groundwater environments (Baquero et al., 2008).

The urban wastewaters may not ideally undergo an appropriate treatment, so the receiving environments may be impacted by wastewater discharges and antimicrobial compounds can be present at detectable concentration (Rodriguez-Mozaz et al., 2015). There are many rivers that have high concentrations of antibiotic, ARB, and ARGs in their sediment samples. Most of these rivers are impacted by urban wastewater while their pristine origins have no antibiotic or antibiotic resistant contaminations (Pei et al., 2006).

Agriculturally influenced regions, such as broiler feedlots and fishponds are known as important sources of ARGs in the environment (He et al., 2014; Yu et al., 2016). Researchers at the Arizona State University's Biodesign Institute inspected antibiotic use in shrimp, salmon, catfish, trout, tilapia, and swai, originating from 11 countries. In this study, the 47 antibiotics were assessed and researchers discovered traces of five antibiotics (Done and Halden, 2015). Animal husbandry is a major subscriber to the environmental burden of ARGs. Pig manure, with its abundant and diverse ARGs and sheer volume, is a major source of ARGs (Zhu et al., 2013). A study led by He et al. (2014) suggested investigating molecular signatures of more common ARGs like *tet*(W) and *sul*(1) or the ratio of *tet*(W): *sul*(1) to track probable sources of ARGs in complex aquatic environments. Based on these studies consideration of an array of ARGs on behalf of various classes will be beneficial to tracing anthropogenic sources of ARGs (He et al., 2014).

Another hotspot for the ARGs is constructed a wetland, and ARGs accumulated in constructed wetlands sediments are an imperative source of aqueous ARGs because of the regular release of microbes from sediments to water. The variation in patterns and concentrations of ARGs is greatly dependent on the operational and environmental factors of the constructed wetlands rather than to the pollutant source and the major source of ARGs in the constructed wetland is observed to be domestic sewage. In this regard, an integrated surface flow constructed wetland has been studied by Fang et al. (2017), and *sul*(1)*, sul*(3)*, tet*(A)*, tet*(C)*, tet*(E), and *qnr*(S) was observed. In this study, the microbial species, the presence of ARB, and the amount of absorbed contaminate like antibiotics and metals have been investigated (Fang et al., 2017). In another study, six mesocosm-scale constructed wetland with three flow types (surface flow, horizontal subsurface flow, and vertical subsurface flow) were set up. Based on this study, *sul*(1)*, sul*(2)*, sul*(3)*, tet*(G), *tet*(M), *tet*(O), *tet*(X), *erm*(B), *erm*(C), *cml*(A) and *flo*(R) were observed in the wetlands (Chen et al., 2016).

Healthcare centers and hospitals are most important facilities with regard to antibiotic consumption and they are sources of ARB and ARGs (Devarajan et al., 2015; Rowe et al., 2017). Many ARGs including *tet*(M), *tet*(O), *tet*(S), *tet*(Q), *tet*(W), and *mec*(A) have been identified in microbial communities of hospital wastewaters due to the wide consumption of human antibiotics in the environments of the hospital (Zhang et al., 2009). The study on three hospital wastewaters showed that fluoroquinolones (among antibiotic families), and *bla*_*TEM*_*, qnr*(S), *erm*(B), *sul*(1) and *tet*(W) (among ARGs) were detected at the highest concentration (Rodriguez-Mozaz et al., 2015). Devarajan et al. (2015) studied fate of a WWTP effluent that was receiving a wastewater from quite a few health care centers like Centre Hospitalier Universitaire Vaudois which is one of the biggest and the most significant facilities. The effluent of this WWTP was discharged in Lake Geneva and qPCR quantification of fecal indicator bacteria and ARGs in sediment of that lake showed that the average of total bacterial load is 2.75 × 10^11^ copy number per each gram of dry sediment, and the relative abundance of *bla*_*CTX*−*M*_ and *bla*_*SHV*_ in core samples, top layers samples, and surface of core samples were 1.97 × 10^−3^, 1.30 × 10^−3^, and 3.64 × 10^−6^. The reason for these high concentrations of ARGs is related to continuing medical usage of antibiotics like penicillin and aminoglycosides (Devarajan et al., 2015). Wastewater from hospitals is possibly the main source of pathogenic and antibiotic-resistant organisms and as well as the ARGs that are released into the environment. A study was conducted in Oslo city hospitals showed that hydrophobic antibiotics, like tetracycline or ciprofloxacin, were detected in all sludge samples of the hospital and fluoroquinolones were consistently found in hospital effluents (Baquero et al., 2008). The preliminary disinfection of hospital wastewater before its discharge into the sewage system or rivers can prohibit the spread ARGs into the environment.

Many studies aimed at the detection and quantification of ARGs in the aquatic and terrestrial environment like soil, surface waters, constructed wetlands, and WWTPs (Kemper, 2008; Pruden et al., 2013; Fang et al., 2017; Zheng et al., 2017). Among the main sources of ARB and ARGs, more attention should be given to leachates of municipal solid waste landfills. The high amount of antibiotics can dominate in municipal solid waste landfill leachates and may be a source of ARB and ARGs to the environment. The presence of antibiotics, metals, and organic pollutants in municipal solid waste and landfill leachate possibly will intensify the persistence of ARGs (Wu et al., 2015).

### Spread

WHO classified ARB and ARGs as two major threats to public health in the twenty-first century, and spread of ARGs in aquatic ecosystems as an increasing concern (Rodriguez-Mozaz et al., 2015). One of the reasons why ARGs bear great concern is that they are related to mobile genetic elements and can easily pass between microorganisms by horizontal gene transfer (HGT). The HGT is one of the most important mechanisms leading to the distribution of antibiotic resistance in the environment. The transfer can happen from donor bacteria, phages, free DNA, or even from the dead cells to living cells (McKinney and Pruden, 2012; Sharma et al., 2016). There are four different mechanisms of HGT:

- conjugation that is a process at which DNA is transferred from the donor cell to the recipient cell via sexual pilus and requires cell-to-cell contact. Then the recipient cell that was susceptible bacteria previously, become resistant as coded by these freshly acquired resistance genes,
- transformation includes uptake, integration, and functional expression of naked DNA by naturally transformable bacteria,
- transduction that is transfer of DNA from one bacterium into another through bacteriophages,
- and gene transfer agents (GTAs) are bacteriophage-like elements made by several bacteria. GTAs carry random segments of DNA present in the host bacterium, which can be transduced to a recipient cell. GTA particles can be free through cell lysis and spread to a recipient cell (von Wintersdorff et al., 2016).

Aquatic ecosystems are ideal sites for occurrence and spread of ARGs since they are constantly polluted by antimicrobial compounds resulting from anthropogenic activities (Rodriguez-Mozaz et al., 2015). These ARGs accumulate in the ecosystem and transfer to clinical pathogens through HGT, causing the failure of antibiotic treatment in the future (Zhou et al., 2016). A study led by Zhou et al. (2016) explored prevalence of ARGs in dairy farms and detected a variety of ARGs and mobile genetic elements (transposase) in feces and soil samples. The results showed the positive correlation (*p* < 0.001) between the total amount of transposase genes and ARGs and suggested the high mobility of ARGs (Zhou et al., 2016).

Considerable amounts of one type of ARGs, such as class 1 integron gene (*intI1*), may lead to subsequent HGT in the WWTP and the spread and occurrence of ARGs and multi-resistant microorganisms (Du et al., 2015). It has been illustrated through a case study on observing variation of ARGs in municipal WWTP that adopted anaerobic/anoxic/aerobic membrane biological reactor (MBR) (Du et al., 2015). It is notable that different genes encoding for specific antibiotic are often located in the same position of chromosomes or mobile genetic elements which lead to multiple resistances (Xu et al., 2017). Therefore, mobile genetic elements for instance plasmids, transposons, and integrons play a significant role in the emergence and spread of ARGs (Zhu et al., 2013). The transposases mostly belong to a family of insertion sequences and are typically found flanking an array of resistance genes. Integrons contain resistance cassettes encoding different ARGs including aminoglycoside and sulfonamide resistance genes, as well as *qacE*Δ*1* efflux pump genes (Zhu et al., 2013). These multi-gene cassettes can encode different ARGs under a mutual promoter and help co-selection of ARGs, hence, selection pressure applied by one antibiotic may select for ARGs associated with diverse antibiotics within the gene cassette of the integron (Miller et al., 2013; Di Cesare et al., 2016). MRSA is a relevant example of the gaining a gene cassette that results in the transfer of multiple ARG simultaneously (Sharma et al., 2016). Trimethoprim resistance mechanism is also the replacement of a trimethoprim-sensitive dihydrofolate reductase by a plasmid-, transposon-, or cassette-borne trimethoprim-resistant dihydrofolate reductase (Zhang et al., 2009).

Although the spread and distribution of ARGs in different environmental systems are well studied, the ecological properties that could result in the selection of ARGs are lesser known, for example, the presence of heavy metals in the environment can cause co-selection of antibiotic and heavy metal resistance. Typical heavy metals such as Cu and Zn are used widely in industry and play important roles in increasing the abundance of certain ARGs (Li H. et al., 2017). Heavy metals are natural compounds present in different ecosystems and are known as a selective pressure on antibiotic resistance and heavy metals exposure may be responsible for antibiotic resistance in either the absence or the presence of antibiotics themselves (Peltier et al., 2010). Understanding heavy metal resistance in natural ecosystems may help to understand antibiotic resistance in the environment since the molecular mechanisms influencing the selection of genes are similar and there are relations between the occurrence of heavy metal resistance genes (HMRGs) and ARGs (Knapp et al., 2017). The elements involved in the resistance to heavy metals are encoded in the chromosomes of bacteria like *Ralstonia metallidurans* (Mergeay et al., 2003), which is well adapted for surviving in naturally heavy metals-rich habitats (e.g., volcanic soils).

The fate of various ARGs and HMRGs have been studied and the results show that these genes can be divided into two groups. The first group includes genes that co-presence does not hint to their co-occurrence. For example, co-presence of *tet*(A), *qnr*(S) (ARGs), and *ars*(B), *czc*(A) (HMRGs) in WWTPs is just because WWTPs are hotspots of different microbial communities of both gram-positive and gram-negative bacteria. The second group of ARGs and HMRGs is showing a strong correlation to each other. For example, Di Cesare and his co-workers obtained a strong correlation between *sul*(2) and *czc*(A), however, the potential mechanisms of such co-selection were never explored (Di Cesare et al., 2016).

Environmental factors are significant subscribers to any types of ecosystems to the transport and spread of antibiotic resistance in the community (Riquelme et al., 2013). It is important to understand the mechanisms that lead to antibiotic resistance and detect factors that provide selective pressures in wastewater habitats (Rizzo et al., 2013). For example, antibiotics, quaternary ammonium compounds or high concentrations of heavy metals resulted in the selection of class 1 RIs-harboring bacteria (Rizzo et al., 2013). Total organic carbon (TOC) concentrations that is one of the environmental factors can affect the selection for some certain genes [*tet*(A), *erm*(B), and *qnr*(S)]. Hence an effluent of a WWTP with high TOC concentrations can change the magnitude and distribution of ARGs in receiving environments (Di Cesare et al., 2016).

## Treatment strategies

The high amount of ARGs and antibiotics can dominate in WWTPs, landfills, municipal solid waste leachates, the soil of dairy farms, and surface waters. In order to limit the occurrence and spread of antibiotic resistance, treatment methods should be able to destroy ARGs in addition to inactivating pathogens (McKinney and Pruden, 2012). Efforts that have been made to combat ARGs are summarized in Table 3 and are further discussed in this section.

**Table 3:** Removal of ARGs by different treatment processes.

**Target**	**Log removal**	**References**
**ANAEROBIC AND/OR AEROBIC TREATMENT REACTORS**		
*tet*(G), *tet*(W), *tet*(X), *sul*(1), and *intI*(1)	–	Du et al., 2015
*sul*(1), *tet*(W), and *tet*(O)	2.37 to 7.06^a^	Munir et al., 2011
**BIOCHAR**		
*sul* genes	1.21	Ye et al., 2016
**CONSTRUCTED WETLANDS**		
*sul*(1), *sul*(2), *sul*(3), *tet*(G), *tet*(M), *tet*(O), *tet*(X), *erm*(B), *erm*(C), *cml*(A) and *flo*(R)	0.44 to 0.80	Chen et al., 2016
*sul*(1), *tet*(A), *tet*(C), *tet*(E), *qnr*(S), *sul*(1), *sul*(3), *tet*(A), *tet*(C), *tet*(E), and *qnr*(S)	0.39 to 0.65 removal rates of total 14 targeted ARGs	Fang et al., 2017
**DISINFECTION**		
*tet*(C), *tet*(G), *tet*(W), *tet*(X), *sul*(2), *drfA1, drfA7, erm*(B), *erm*(F), *erm*(Q), and *erm*(X)	0.1 to 2.3	Li H. et al., 2017
*ere*(A), *ere*(B), *erm*(A), *erm*(B), *tet*(A), *tet*(B), *tet*(M), and *tet*(O)	0.42 and 0.10 removal of *erm* and *tet* genes, respectively	Yuan et al., 2015
*sul*(1), *tet*(X), *tet*(G), *intI*(1), and 16S rRNA	1.30 to 1.49	Sharma et al., 2016
**COAGULATION**		
*sul, tet*, and integrase genes	0.5 to 3.1	Li N. et al., 2017

a *Conventional treatment plants and MBR facility*.

### Anaerobic and/or aerobic treatment reactors

Aerobic and anaerobic treatment processes are low energy and environmentally friendly strategies which are mostly used to treat chemical oxygen demand (COD), moreover, they can successfully remove ARB and ARGs (Christgen et al., 2015). The aerobic treatment processes occur in the presence of air and microorganisms which use oxygen to convert organic contaminants to carbon dioxide, water, and biomass (aerobes). The anaerobic treatment processes, on the other hand, take place in the lack of air and microorganisms which do not require air to convert organic contaminants to methane and carbon dioxide gas and biomass (anaerobes) (Grady et al., 1999).

Samples of a municipal WWTP has been studied to evaluate the variation of five ARGs [*tet*(G), *tet*(W), *tet*(X), *sul*(1), and *intI*(1)] in the influent and effluent of each treatment unit. The WWTP possessed the anaerobic/anoxic/aerobic MBR process. The concentration of ARGs in wastewater diminished in the anaerobic and anoxic effluent, while increment in the aerobic effluent was observed. Later the ARGs concentration declined in the MBR discharge. Based on this study, it was concluded that anaerobic and anoxic treatments are much more successful to remove ARGs rather than aerobic treatment since microorganism has lower bioactivity under anaerobic condition and the propagation of resistance genes are inhibited (Du et al., 2015). There was significant positive correlation observed between the reduction of *tet*(W), *intI*(1), and *sul*(1) and the reduction of 16S rDNA in the wastewater treatment process (Du et al., 2015).

Anaerobic–aerobic sequence (AAS) bioreactors also is a low energy treatment option including an anaerobic treatment to diminish carbon concentration as a pretreatment and then aerobic treatment. Metagenomics studies of this treatment method showed the effect of this approach on antibiotic resistance in general and ARG in particular. AAS removed more than 85% of ARGs in the influent which means it was more efficient compared with aerobic and anaerobic units (83 and 62%, respectively; Christgen et al., 2015).

In another study, occurrence and release of tetracycline-resistant and sulfonamide-resistant bacteria, as well as three genes [*sul*(1), *tet*(W), and *tet*(O)] in the effluent of five WWTPs was studied, and performance of different processes was compared. ARGs and ARB removal ranged 2.57-log to 7.06-log in MBR, and 2.37-log to 4.56- log in activated sludge, oxidative ditch and rotatory biological contactors (Munir et al., 2011).

Removal of antibiotics including sulfamethazine, sulfamethoxazole, trimethoprim, and lincomycin had been studied in five different WWTPs using aerobic/anaerobic treatment methods (Behera et al., 2011). It was found that the removal efficiency of antibiotics was low compared with other pharmaceutical compounds. The removal efficiency of sulfamethazine, sulfamethoxazole, trimethoprim, and lincomycin was 13.1, 51.9, 69.0, and −11.2%, respectively. High load of lincomycin led to its negative removal efficiency (Behera et al., 2011).

To sum it up, biological treatment methods can remove antibiotics, ARB, and ARGs successfully if anaerobic and aerobic reactors operate in sequence, and aerobic reactors alone are not effective. If membrane-based technologies, like MBR, can be used in combination with biological treatment the better ARG removal efficiency would be achieved.

### Constructed wetlands

Constructed Wetlands are small semi-aquatic ecosystems, in which a great population of different microbial community multiplies and various physical-chemical reactions happen. Over the past years, man-made wetlands have been designed and they are known as attractive municipal, industrial and agricultural wastewater treatment approaches because of their simplicity, cost efficiency, and effect on eliminating ARGs (Fang et al., 2017). Characteristics of constructed wetland can affect ARB and ARGs removal efficiency. These characteristics are namely, flow configuration, plant species and flow types including (surface flow, horizontal subsurface flow, and vertical subsurface flow). Biodegradation, substrate adsorption, and plant uptake all play a certain role in decreasing the loadings of nutrients, antibiotics, and ARGs in the constructed wetlands, however, biodegradation is the most vital process in the removal of these pollutants (Chen et al., 2016).

Constructed Wetlands can efficiently remove aqueous ARGs however they can also act as reservoirs for specific ARGs. A study led by Fang et al. (2017) suggested constructed wetland as a domestic sewage treatment method. They attained 77.8 and 59.5% removal rates of total 14 targeted ARGs in the integrated surface flow constructed wetlands in the winter and summer season, respectively. The results of this study also found strong positive correlations between concentrations of intI1 and ARGs, indicating that mobile genetic elements affect the dissemination of ARGs in a constructed wetlands (Fang et al., 2017).

The removal of ARB and ARGs in raw domestic wastewater by differently constructed wetland has been investigated and 8 antibiotics and 12 genes, and 16S rRNA (bacteria) were studied in different matrices. The aqueous removal efficiencies of total antibiotics ranged from 75.8 to 98.6%, while those of total ARGs fluctuated between 63.9 and 84.0% by the constructed wetland. The presence of plants was beneficial to the removal of pollutants, and the subsurface flow constructed wetland had higher pollutant removal than the surface flow constructed wetlands, particularly for antibiotics (Chen et al., 2016).

### Disinfection

Disinfection of water and wastewater is a process that kills a significant percentage of pathogenic organisms that may cause bacterial, viral or parasitic diseases. The most popular disinfection process in wastewater treatment is chlorination since it is available and effective, however, ozone and UV radiation also are employed. WWTPs are hotspots for ARB and ARGs, and there are so many heterotrophic bacteria in the effluents exhibiting resistance to multiple antibiotics (Pang et al., 2016). Effective disinfection is a vital and regular process for disruption of ARB and ARGs and inactivation of harmful microorganisms.

Effect of Chlorination on the removal of different antibiotics such as cephalexin, ciprofloxacin, chloramphenicol, erythromycin, gentamicin, rifampicin, sulfadiazine, tetracycline, and vancomycin have been previously monitored along with its effect on inactivation of ARB and ARGs. Advanced oxidation processes utilizing ozone, UV irradiation, Fenton reagent, and photocatalytic systems have also been applied to disinfection process. Some studies were also extended to two or more processes in regard to making a comparison of their efficacy and mechanism (Keen and Linden, 2013; Sharma et al., 2016).

Yuan et al. (2015), studied fates of nine different ARB and two series of ARGs [*ere*(A), *ere*(B), *erm*(A), *erm*(B), *tet*(A), *tet*(B), *tet*(M), and *tet*(O)] in treated wastewater using chlorination. Their detailed quantitative real-time PCR examination and analysis showed 60 and 20% removal of these genes, respectively, applying various doses of chlorine ranging from 15 to 300 $\frac{mg\;Cl_{2}}{min.\;L}$. All the bacteria, other than sulfadiazine- and erythromycin-resistant bacteria, were inactivated fully by just 15 $\frac{mg\;Cl_{2}}{min.\;L}$. Sulfadiazine- and erythromycin-resistant bacteria were inactivated when the chlorine dose was more than 60 $\frac{mg\;Cl_{2}}{min.\;L}$ (Yuan et al., 2015).

A recent study explored three disinfection methods including chlorination, single UV irradiation, and sequential UV/chlorination to compare their efficiency to combat ARGs in municipal WWTP (Sharma et al., 2016). ARGs including *sul*(1), *tet*(X), *tet*(G), *intI*(1), and 16S rRNA were considered and the results proved a positive relationship between the inactivation of ARG and the parameters involving dosage of chlorine, contact time, and the maximum inactivation. Based on the results, maximum inactivation was in the range from 1.30-log to 1.49-log at 30 $\frac{mg}{min.\;L}$ of Cl_2_ (Sharma et al., 2016). Single UV irradiation was less effective while sequential UV/chlorination had the maximum ARGs removal efficiency.

A similar lab-scale research was established to investigate the inactivation of *sul*(1), *tet*(G), and *intI*(1) by three different disinfection methods involving Chlorination, UV irradiation, and Ozonation. Samples for lab test were obtained from a municipal WWTP effluent and gene copies of *sul*(1), *tet*(G), *intI*(1), and 16S rDNA was measured (Zhuang et al., 2015). Results from this study suggest that chlorination was most effective in inactivation of ARGs compared to other methods (Zhuang et al., 2015).

### Nanomaterial

Many diverse combinations of nanomaterial have proved that antimicrobial nanotechnology can be effective defenses against antibiotic resistance, ARB, and ARGs. Two different mechanisms are probable when nanoparticles treat antibiotic resistance; in the first mechanism a functionalized nanomaterial is combined with antibiotics and nanomaterial enters inside ARB and then release considerable amounts of toxic ions. In the second mechanism, a combination of antibiotic and nanomaterials result in synergistic effects, that is they combat ARGs separately (Aruguete et al., 2013).

The potential for antimicrobial nanomaterials to restrict the propagation of multi-drug resistant pathogens while avoiding the generation of new nanomaterial-resistant organisms was studied by a group of researchers led by Aruguete et al. (2013). They prepared a combination of nanomaterials functionalized with molecular antibiotics. This combination consisted of liposomes, dendrimers, and an antibiotic that is inside of a polymer nanoparticles capsules, and inorganic nanoparticles with antibiotic molecules attached to the surfaces (Aruguete et al., 2013). In this study, silver nanoparticles coated with a water-soluble polymer called polyvinylpyrrolidone were used to combat nanomaterial-resistant organisms (Aruguete et al., 2013). This experiment proved that nanomaterial combinations are able to perform like an antibiotic and are toxic to *Pseudomonas aeruginosa* bacteria which was resistant to multiple drugs (Aruguete et al., 2013).

Nanomaterials have been considered as a defense against multiple drug resistance because of their antimicrobial activity (Shahverdi et al., 2007; Monteiro et al., 2009; Lam et al., 2016; Yu et al., 2016). Antibacterial activities of nanoparticles depend on two fundamental elements, physicochemical properties of nanoparticles and type of target bacteria. Despite the fact that there is a great correlation in a few aspects of the antibacterial activity of nanoparticles, individual studies are challenging to generalize because the majority of researchers perform experiments in light of accessible nanoparticles and bacteria, instead of targeting particular and preferred nanoparticles or bacteria (Hajipour et al., 2012). Nanoparticles which are used in lab-scale studies are not well-known and correlating them with basic physicochemical properties for full-scale production is difficult.

Recently, a mixture of nanomaterials and molecular antibiotics attracts much attention since they are effective in killing multi-drug resistant isolates of pathogenic bacterial species and combating a broad spectrum of ARB and ARGs (Aruguete et al., 2013; Sharma et al., 2016). A few studies in regard to nanoparticles and their role in combating ARB and ARG are summarized in Table 4.

**Table 4:** Nanoparticles combating ARB and ARGs.

**Types of Nanoparticles**	**Source**	**Target**	**References**
Nanosilver and sulfamethoxazole	Sulfamethoxazole Anaerobic Digester Sludge	ARGs: *tet*(O), *tet*(W), *sul*(1), *sul*(2), and *intI*(1)	Miller et al., 2013
Silver nanoparticles	An aquatic environment with Fe^3+^ or Fe^2+^ ions and natural organic matter	ARB: *Enterococcus faecalis* *Staphylococcus aureus* *Staphylococcus epidermidis* *Pseudomonas aeruginosa* *Klebsiella pneumoniae*	Adegboyega et al., 2014
Silver	Aqueous solution	ARB: *Mycobacterium tuberculosis* Multi-drug resistant *S. pneumoniae*	Singh et al., 2014
Nitric oxide releasing nanoparticles	Aqueous solution	ARB: *Acinetobacter baumanii methicillin-resistant S. aureus* *Klebsiella pneumoniae* *Pseudomonas aeruginosa*	
Silver nanoparticles with NOM and Iron	Aqueous solution	ARB: methicillin-resistant *S. aureus*	Sharma et al., 2014
Nanoparticles including copper oxide (CuO), zinc oxide (ZnO), and TiO_2_	Aqueous environment	ARB: *E. coli* and *B. subtilis*	Pavithra et al., 2015
Ceftriaxone- ZnO Nanorods	Aquatic solutions of E. coli with ceftriaxone, ZnO nanorods and ceftriaxone-ZnO nanorods with phosphate-buffered solution	ARB: *E. coli*	Luo et al., 2013
Superparamagnetic iron oxide nanoparticles (conjugation of iron, zinc, and silver)	Treatment of medical device infections	ARB: multi- drug resistant *S. aureus* and antibiotic-resistant biofilms ARGs: *Staphylococcus*	Taylor et al., 2012
Nano alumina	Water	ARB: *E. coli* and *Salmonella* spp.	Qiu et al., 2012
Gold nanoparticles with vancomycin	Aqueous solution of polyvinyl alcohol	ARB: vancomycin resistant *S. aureus, E. coli* strain	Mohammed Fayaz et al., 2011
Iron oxide nanoparticles	Aqueous solution	ARB: *S. aureus*	Tran et al., 2010

Nanomaterials play controversial roles regarding antibiotic resistance; on one hand, as mentioned before, they have been considered as a defense against multiple drug resistance because of their antimicrobial activity. On the other hand, nanomaterials can encourage the development of antibiotic resistance in the environment (Aruguete et al., 2013; Miller et al., 2013) and also some of them have shown toxic effects on fauna, flora, and human beings, such as infection, cytotoxicity, tissue ulceration, and reduction of cell capability (Srivastava et al., 2015). In this regard, silver nanoparticles which are tremendously used for its application in drug delivery, like Citrate-coated silver nanoparticles, demonstrated genotoxicity and cytotoxicity *in vitro* and *in vivo*. Besides, iron and iron oxide nanoparticles are widely used because of their magnetic target drug delivery potential, however, their *in vitro* cytotoxicity has been established (Srivastava et al., 2015).

Overall, more information is needed concerning the mechanisms behind the antimicrobial activity of nanomaterials and their potential for influencing the development of resistance and their toxic effect on the proper microflora of water or soil and more importantly on plants, animals, and humans.

### Coagulation

Coagulation is an active method to remove colloidal particles in water and treat turbidity, color, natural organic matter, and heavy metals (Zainal-Abideen et al., 2012). Colloidal particles mostly have negative electrical charges while coagulants carry a positive charge. A coagulant neutralizes particles and makes them stick together when contact is made. The coagulation process as the tertiary treatment process in WWTPs is broadly utilized for improving water quality and removing contaminants.

Different types of organic and inorganic coagulants have been prepared based on the target contaminants. A study has been conducted to treat drinking water and assess removal of the natural organic matter by zirconium coagulant (Jarvis et al., 2012). Zirconium coagulant has improved water quality and provided lower dissolved organic carbon residual rather than iron coagulant (Jarvis et al., 2012). Furthermore, the flocs formed by using zirconium coagulant were larger and stronger compared to iron and aluminum salts coagulant. For example, floc sizes were 930, 710, and 450 μm for zirconium, iron and aluminum coagulant, respectively (Jarvis et al., 2012). Phosphorus contamination also can be removed by coagulation. Residual phosphorus in the effluent of WWTPs can result in eutrophication and affect the environment. A research has been directed that used electrochemical coagulation and to remove phosphorus. The results showed 97% phosphorus removal regardless of initial concentration (Tran et al., 2012). A study on the coagulation behavior of polyferric chloride showed that it can influence density, the stability of flocs and improve removal of algal cell, turbidity, color, and humic acid in eutrophicated water (Lei et al., 2009). Persistent organic pollutants in surface water, like perfluorooctane sulfonate and perfluorooctanoate, can be removed from drinking water by using alum and ferric chloride as a coagulant (Xiao et al., 2013). Solution pH, coagulant dosage, coagulants, natural organic matter concentration, turbidity, and flocculation time can affect the removal efficiency (Xiao et al., 2013).

In recent years, the effectiveness of coagulation technology in the removal of ARGs from treated wastewater has been investigated Li N. et al. (2017). Coagulation is an active method for ARG removal from WWTP effluent. Li H. et al. (2017) used inorganic coagulant FeCl_3_ and inorganic polymer coagulant poly ferric chloride (PFC) and examined the removal of sul genes, tet genes, and integrase genes. Significant removal correlations were detected between dissolved NH_3_-N and DOC^1^ and ARGs. ARGs removal efficiency ranged from 0.5-log to 3.1-log reductions (Li N. et al., 2017).

### Biochar

Biochar is active charcoal derived from the pyrolysis of carbon-rich biomass. Biochar is a porous material that has rich mineral elements and large specific surface area (Ye et al., 2016), and thus provide the most sites to be filled by sorption of contaminants.

IUPAC^2^ classification categorized the porosity of biochar into micropores (<2 nm), mesopores (2–50 nm), and macropores (>50 nm) (Gray et al., 2014). The main treatment mechanism of biochar is sorption and base on the properties of contaminant, micropore-filling or macropore-filling would dominant for sorption on biochar. Moreover, electrostatic repulsion is another sorption mechanism that is attributed to complex properties of biochar. Different parameters including various proportions of carbon and inorganic fractions, pyrolytic temperature, and source of a compound that are used to prepare biochar would affect properties of biochar (Zheng et al., 2013).

The effect of biochar on ARGs has been studied recently and based on the results significant change in the microbial communities was observed after addition of biochar in the soil. Various types of biochar prepared from different feedstocks cause different changes in microbial community structures. Change in bacterial phylogenetic compositions can result in a change of ARGs, and thus the addition of different biochar to the manure composting may have the effects on the relative abundance of ARGs (Cui et al., 2016).

Biochar amendment has been recently studied to assess its effects on preventing antibiotic, ARB, and ARGs from accumulating in tissues of vegetable that was cultivated in contaminated soil. Ye et al. (2016) conducted experiments in pots and cultivated lettuce on a soil contaminated by sulfonamides. They concluded that lettuce cultivation with biochar amendment can dissipate sulfonamides from soil and offer the highest growth indices. Furthermore, the concentration of sulfonamides and bacterial endophytes resistant to sulfonamides in roots and leaves were reduced by one to two orders of magnitude compared to vegetables grown without biochar amendment (Ye et al., 2016).

In a research study, antibiotic removal has been studied using biochars produced at different temperatures. The K_d_ for the sorption of sulfamethoxazole was found to be ranging from 30 to 1,675 L kg^−1^ showing that biochar has the potential for remediation of soil or water containing sulfonamides (Zheng et al., 2013).

Overall, using biochar is a practical strategy that can treat antibiotics contaminated soils and also prevent antibiotics, ARB, and ARGs from accumulating in the vegetation (Ye et al., 2016).

## Conclusion and recommendation

The accelerating antibiotic resistance development among bacteria is a challenging issue that requires improvement of next-generation treatment processes. It has been observed that Low-energy anaerobic–aerobic treatment reactors reduce high concentrations of various ARGs from domestic wastewater. Over the past years, constructed wetlands with different flow configurations or plant species have been designed and they are known as attractive wastewater treatment approaches on eliminating ARGs from raw domestic wastewater. Most of the studies on the inactivation of ARG by disinfection have been conducted using chlorination, however, some studies were also extended to ultraviolet irradiation, allowing an evaluation of the two processes regarding their efficacy and mechanism. Recently, nanoparticles are known as antimicrobial agents that are effective to remove ARG and are known as a novel defense against ARB when accompanied by antibiotics. The coagulation process as the tertiary treatment process in WWTPs has recently known as an active method for ARGs removal and the use of biochar makes a notable change in the microbial communities and inactivate ARGs after addition of biochar amendment in the soil.

The emergence of antibiotic resistance among pathogens increases the demand for novel treatment strategies. Even though significant efforts have been made to investigate the treatment methods combating ARB and ARGs, there are considerable gaps to fill in:

- Studies have mainly been lab-scale or pilot-scale and over short operation time. It is important to conduct large-scale testing on real samples derived from the environment.
- Develop risk assessment studies to estimate precise values of the abundance of ARB and ARGs in WWTP discharges that do not trigger human health issues.
- Considering the effect of the operating conditions (pH, free available chlorine, HRT, SRT, Biomass concentration), environmental factors (temperature, COD, BOD, water flow), and mechanisms (mutation, selection, mechanisms of genetic exchange including conjugation, transduction, and transformation) that may increase antibiotic resistance bacteria and genes while treating water or wastewater in plants.
- Assessing the feasibility of implementing modifications to improve existing water and wastewater treatment facilities to increase ARBs and ARGs in the effluent of plants.
- Conduct further studies to determine the fate of ARBs and ARGs that encode the more extensive spectrum of antibiotics namely fluoroquinolone, ertapenem, and levofloxacin resistance in municipal wastewater.
- Future studies on the effect of stress conditions on ARG horizontal transfer and fate of ARGs at anaerobic digesters and aerobic reactors.
- Consider seasonal changes in ARB and ARGs amounts and perform experiments over sufficient time while using constructed wetlands as a treatment method. Exact factors controlling ARG levels and patterns in sediments of the constructed wetland system must be studied since they affect variation and patterns of ARGs.
- Investigating advanced treatment systems and combined disinfection methods to discover a suitable and cost-effective method to remove ARGs from WWTP effluents. Since the current disinfection units at WWTPs can only partially remove ARGs.
- Gain more information to figure out the mechanisms behind the antimicrobial activity of nanomaterials and their probable effect on the increase of resistance in the environment. For example, molecular mechanisms causing antimicrobial activity in nanomaterials and microbial interactions with nanomaterials should be studied to prevent the development of microbial resistance nanomaterials.
- Current practices ensured that some coagulants partially eliminate ARGs, however, further removal of ARGs from WWTPs should be examined because coagulation is an essential part of treatment plants and their potential to treat ARB and ARGs would be practical for many existing plants.
- Case studies on using biochar for treating water and wastewater, for example, evaluating filtration applications of biochar.

In light of all above-mentioned gaps in treatment strategies to combat ARB and ARGs, conducting further research in this area is essential to provide the opportunity to increase the efficiency of existing strategies or innovate new approaches.

## Author contributions

FB and MM conceived, designed and formulated the outline of this manuscript. FB wrote the manuscript and MM provided feedback and discussion on the manuscript.

### Conflict of interest statement

The authors declare that the research was conducted in the absence of any commercial or financial relationships that could be construed as a potential conflict of interest.

## Footnotes

^1^Dissolved organic carbon.

^2^International Union of Pure and Applied Chemistry.
